# Dietary Protein Intake and Peritoneal Protein Losses in Peritoneal Dialysis Patients

**DOI:** 10.1002/1744-9987.70146

**Published:** 2026-04-15

**Authors:** Haalah Shaaker, Andrew Davenport

**Affiliations:** ^1^ King Abdulaziz University Jeddah Saudi Arabia; ^2^ Division of Medicine University College London London UK; ^3^ Centre for Kidney & Bladder Health, Royal Free Hospital University College London London UK

**Keywords:** bioimpedance, body composition, dietary protein intake, peritoneal dialysis, protein losses

## Abstract

**Introduction:**

Peritoneal dialysis (PD) patients lose protein in their waste dialysate, potentially increasing their risk for malnutrition. We wished to determine whether there was any association between losses and dietary protein intake (DPI).

**Methods:**

DPI was assessed from 24‐h dietary recall using Nutrics software. Total protein losses were measured in 24‐h collections of spent dialysate and urine.

**Results:**

We studied, 41 patients, mean age 62.2 ± 15.8 years, 56.1% male, treated for 14.0 (3–23) months. Only13 (31.7%) met the recommended DPI target of ≥ 1.0 g/kg/day, and they had greater peritoneal protein losses 5.2 ± 2.4 versus 3.3 ± 1.1 g/day, *p* < 0.001, and faster peritoneal solute transfer rates (PTSR ×100 for protein 0.93 (0.58–1.21) versus 0.44 (0.35–0.56), *p* < 0.001). On multivariate analysis DPI was associated with calorie intake (standardized beta coefficient (Stꞵ) 0.56, *p* < 0.001, and peritoneal protein clearance rate (Stꞵ 0.36, *p* = 0.002).

**Conclusion:**

Patients with faster protein clearance and protein losses had higher DPI.

## 1 Introduction

There is a gradual and progressive loss of muscle mass along with reduced strength and increasing risk of frailty with aging [1]. This has led to suggestions that dietary protein intake (DPI) be increased above the recommended dietary allowance of 0.8 g/kg/day, with both observational and interventional studies demonstrating that participants and patients achieving a higher DPI were more likely to maintain or increase muscle mass compared to those with lower DPI [2]. As patients with end‐stage kidney disease (ESKD) may also have losses of protein with dialysis treatments, clinical guidelines recommend a higher DPI of 1–1.2 g/kg/day [3].

Protein intake may be the result of the physiological response to acute or chronic changes in energy balance, but also eating for pleasure, so‐called hedonic eating [2]. However, dietary intake may be reduced in ESKD patients due to many causes, including altered taste perception, dietary restrictions, and for patients treated with peritoneal dialysis (PD) the physical presence of intra‐abdominal dialysate, coupled with glucose absorption from the dialysate and protein losses into the dialysate [4, 5].

Over time, the demographics of the PD dialysis population have changed in high‐income countries with the introduction of automated peritoneal cyclers (APD) and assisted PD programs, leading to greater numbers of older, more co‐morbid ESKD patients now being offered PD. As reports in the general elderly population have shown an association between DPI and muscle mass [6], we wished to determine whether PD patients with a higher DPI had greater muscle mass.

## 2 Methodology

### 2.1 Study Population

Forty‐one established adult PD outpatients attending for routine assessments of peritoneal membrane testing under the care of a university hospital were studied. Patients unable to provide accurate dietary recall, due to cognitive impairment, physical disability, or language barriers were excluded. Patients who had acute hospitalizations or episodes of peritonitis in the preceding 3 months were also excluded. No patient was prescribed steroids or glucose transporter inhibitors. All patients used glucose‐based PD dialysates and 7.5% icodextrin (Baxter Health Care, Deerfield, Illinois, USA) for overnight exchanges with continuous ambulatory peritoneal dialysis (CAPD) or daytime exchanges with automated continuous cycling PD (CCPD).

### 2.2 Assessments

#### 2.2.1 Dietary Intake

A 24‐h dietary recall was conducted by a single trained dietitian to estimate nutrient intake, and then analyzed using Nutritics software (Nutrias, Swords, County Dublin, Ireland) [7]. Patients were also asked to score their appetite and food satisfaction and thirst using a visual analogue score [8, 9].

#### 2.2.2 Body Composition and Muscle Strength

Body composition was measured using multifrequency bioelectrical impedance analysis (BIA), following a standard protocol, after patients had voided and peritoneal dialysate drained (InBody 770, Seoul, South Korea) [10]. Bioimpedance equipment was regularly serviced and calibrated. To compare patients, the appendicular lean mass index (ALMI) was calculated by dividing appendicular lean mass (ALM) by height squared. Hand grip strength (HGS) was measured with a Grip‐D dynamometer (Takei 5401 Hand Grip Digital Dynamometer, Shinagawa‐ku, Tokyo, Japan) to determine muscle power, with patients instructed according to the manufacturer's instructions, with the maximum of three measurements recorded [11]. The Duke activity status index (DASI) questionnaire was used to assess functional capacity [12].

#### 2.2.3 Clinical Assessments

Co‐morbidity was scored according to Stoke‐Davies [13], and frailty using the Rockwood clinical frailty score [14]. The presence of sarcopenia was defined according to the European Working Group on Sarcopenia (EWGSOP) criteria for non‐Asians (HGS < 27 kg for men, < 16 kg for women, and ALMI < 7 kg/m^2^ for men, < 5.5 kg/m^2^ for women), and the Asian Working Group for Sarcopenia for Asian patients (HGS of < 28 kg and < 18 kg, and ALMI < 7.0 kg/m^2^ and < 5.7 kg/m^2^ for Asian men and women, respectively) [15, 16].

#### 2.2.4 Dialysis Treatment

Dialysis adequacy (*K*_*t*_/*V*_urea_) was determined from corresponding 24‐h collections of drained dialysate and urine and the peritoneal solute transfer rate (PSTR) using a 4‐h dwell of a 2.0 L 22.7 g glucose/L dialysate. Peritoneal dialysate and urinary protein losses were measured turbidimetrically using the benzethonium chloride method [17], and glucose absorption calculated from the amount of glucose instilled in fresh dialysate and that recovered from spent dialysate [18]. Standard biochemical tests were all measured by an accredited United Kingdom (UK) laboratory, and dialysis adequacy and dietary protein estimated as protein nitrogen appearance rate (PNA) using standard formulae [19, 20].

### 2.3 Ethics Approval

The study was approved by the UK National Health Service (NHS) Research Authority Ethics Service (NRES) (23/PR/0787). In keeping with the Helsinki accord, all patients provided written informed consent, and following NRES protocols all data were anonymized.

### 2.4 Statistical Analysis

Data normality was assessed using skewness and kurtosis. Categorical variables were analyzed using chi‐squared tests, and numerical data presented as mean ± standard deviation or median (interquartile range). Comparisons were made using independent *t*‐tests or Mann–Whitney U tests, with appropriate post hoc testing. Univariate correlations were evaluated with Pearson or Spearman correlation coefficients. Variables associated with diet recall DPI on univariate analysis were entered into a step backward multivariable regression model to determine which variables were independently associated with DPI. If required variables were log transformed to improve normality distribution. Variables were then removed or retained in the model if the 95% confidence intervals for the estimate did not include zero or there was an improvement in model fit (as demonstrated by the −2 log likelihood). Models were checked for collinearity and variable inflation factor to prevent overloading and checked for collider bias. Analyses were performed with IBM SPSS Statistics Version 29 (IBM, Armonk, New York, USA) and Prism (Graph Pad Prism 10.6.1, Prism, San Diego, USA) with significance taken at *p* < 0.05.

## 3 Results

We studied 41 patients, and 13 (31.7%) met the recommended DPI target of ≥ 1.0 g/kg/day, and 12 (29.3%) the calorie target of 25–30 kcal/kg/day. Although not significantly different, patients who met DPI targets were marginally older, with fewer men and diabetics (Table 1). Similarly, there were no differences in frailty or co‐morbidity scores, echocardiography ejection fraction, or physical activity using the DASI tool. Residual kidney function was similar for both groups, and although not significantly different, patients with lower DPI had been treated with PD for slightly fewer months, but used more hypertonic glucose dialysates and automated peritoneal dialysis (APD). However, patients achieving DPI targets had greater daily peritoneal protein losses and faster PSTR for protein. Standard laboratory testing was not different between groups (Table 2).

**TABLE 1:** Patient demographics and peritoneal dialysis prescriptions and assessments.

	All patients	DPI < 1.0 g/kg/day	DPI ≥ 1.0 g/kg/day
Number	41	28	13
Age years	62.2 ± 15.8	60.7 ± 15.3	65.5 ± 17.0
Male (%)	23 (56.1)	17 (60.7)	6 (46.2)
Weight (kg)	72.7 ± 17.6	74.8 ± 17.8	68.4 ± 16.8
BMI (kg/m^2^)	26.8 ± 5.0	27.8 ± 5.2	24.7 ± 3.9
Diabetic (%)	22 (53.7)	16 (57.1)	6 (46.3)
Frailty score	3 (3–4)	3 (3–4)	3 (3–5)
Davies co‐morbidity	1 (1–2)	1 (1–2)	1 (0–1)
DASI score	23.5 (15.5–38.8)	27.3 (15.5–38)	20.7 (14.1–42.3)
MAP (mmHg)	93.8 ± 11.8	95.7 ± 10.3	89.6 ± 14.6
Ejection fraction (%)	55.5 ± 8.3	55.6 ± 6.6	55.4 ± 11.8
Months PD	14.0 (3–23)	12.5 (3–17.8)	22 (9–48)
CAPD/APD/CCPD (%)	36.6/17.1/46.3	28.6/17.9/53.6	53.9/15.4/30.8
Icodextrin (L/day)	1.5 (0.7–2.0)	1.35 (0.73–1.88)	2.0 (0.35–3.25)
22.7 g glucose/L (L/day)	2.0 (0–8.9)	4.0 (0–11.3)	1.0 (0–4.7)
Dialysate (L/day)	10 (3.5–13.2)	11.2 (4.0–13.2)	6.5 (3.3–12.5)
Urine volume (mL/day)	908 (183–1763)	802 (109–1769)	1020 (833–1809)
GFR ml/min/1.73 m^2^	4.4 (1.3–7.5)	4.3 (0.9–8.5)	4.4 (3.0–5.9)
Weekly (*K*_*t*_/*V*_urea_)	2.17 (1.73–2.55)	2.16 (1.57–2.58)	2.19 (1.91–2.6)
L creatine/wk/1.73 m^2^	71.1 (51.5–89.4)	68.6 (40.3–90.1)	72.4 (61.8–86.4)
UF volume ml/day	800 (320–1257)	872 (342–1372)	640 (252–1148)
Glucose absorption (mmol/day)	166 (17.3–311)	178 (225–384)	92 (−2.9 to 274)
PD protein loss (g/kg/day)	4.0 ± 1.9	3.3 ± 1.1	5.2 ± 2.4 **
PD protein clearance mL/min/1.73 m^2^	0.028 ± 0.015	0.022 ± 0.007	0.039 ± 0.022**
Urine protein (g/day)	0.32 (0.08–0.93)	0.28 (0.01–1.08)	0.33 (0.2–0.79)
PSTR creatinine	0.71 ± 0.14	0.67 ± 0.14	0.69 ± 0.13*
PSTR protein ×100	0.52 (0.38–0.88)	0.44 (0.35–0.56)	0.93 (0.58–1.21)***

*Note:* Patients divided according to dietary recall dietary protein intake (DPI). Data displayed as integer, percentage, mean ± standard deviation, or median (interquartile range). *p < 0.05, **p < 0.01, ***p < 0.001 versus low dietary protein intake.

Abbreviations: APD, ambulatory peritoneal dialysis without a day time exchange; BMI, Body mass index; CAPD, continuous ambulatory peritoneal dialysis; CCPD, ambulatory peritoneal dialysis with a daytime exchange; DASI, Duke activity status index; GFR, glomerular filtration rate; MAP, mean arterial pressure; PD, peritoneal dialysis; PSTR, peritoneal solute transfer rate; UF, ultrafiltration volume.

**TABLE 2:** Patient laboratory investigations.

Laboratory results	All patients	DPI < 1.0 g/kg/day	DPI ≥ 1.0 g/kg/day
Hemoglobin (g/L)	116.8 ± 11.8	118.8 ± 10.9	112.5 ± 12.9
Albumin (g/L)	37.4 ± 5.1	37.9 ± 5.3	36.2 ± 4.5
C reactive protein (mg/L)	3 (1–10)	3 (2–11.8)	2 (1–4.5)
Sodium (mmol/L)	135.5 ± 5.1	136.1 ± 5.6	134.3 ± 3.7
Potassium (mmol/L)	4.4 ± 1.0	4.1 ± 0.7	4.9 ± 0.9
Urea (mmol/L)	19.9 ± 6.1	19.5 ± 5.3	20.8 ± 7.6
Creatinine (μmol/L)	639 (571–817)	656 (570–867)	611 (553–795)
Phosphate (mmol/L)	1.67 ± 0.4	1.68 ± 0.41	1.63 ± 0.39
HbA1c (mmol/mol)	48.2 ± 17.2	48.5 ± 15.9	47.7 ± 20.6
Cholesterol (mmol/L)	5.0 ± 1.2	5.1 ± 1.2	4.8 ± 1.3
ꞵ2 microglobulin (mg/L)	21.6 (16.9–26)	22.3 (16.9–31.4)	19.9 (17–21.3)
NTproBNP (pg/mL)	2136 (812–7194)	2328 (715–8923)	2136 (1029–3460)

*Note:* Patients divided according to dietary recall dietary protein intake (DPI). Data displayed as integer, percentage, mean ± standard deviation, or median (interquartile range). There were no significant differences between higher versus lower dietary protein intake groups.

Abbreviations: HbA1c, glycated hemoglobin; NTproBNP, N terminal probrain natriuretic peptide.

Food satisfaction and thirst stores were similar, but in addition to greater DPI, those patients meeting DPI targets also had greater calorie intake, although the majority of patients failed to achieve the KDOQI guideline target of 25–30 kcal/kg/day [3] (Table 3). However there was no difference in assessments of muscle mass, muscle strength as measured by HGS, patients who achieved DPI targets had lower levels of fat tissue.

**TABLE 3:** Patient self‐reported dietary assessments, and measurements of body composition.

	All patients	DPI < 1.0 g/kg/day	DPI ≥ 1.0 g/kg/day
Appetite score	1 (1–2)	2 (1–2)	1 (0–2)
Food score	4 (2–7)	4.5 (2.3–6)	4.0 (0–7)
Thirst score	4 (4–7.5)	4 (4–7.3)	4 (4–7.5)
Calorie intake (kcal/kg/day)	17.0 (13.3–22.4)	15.6 (12.3–19)	19.6 (15.3–33.2)***
FFMI (kg/m^2^)	16.5 ± 2.6	17.6 ± 13.8	17.3 ± 2.0
LMI (kg/m^2^)	17.5 ± 2.7	16.6 ± 2.9	16.2 ± 1.9
ALMI (kg/m^2^)	7.4 ± 1.6	7.5 ± 1.8	7.2 ± 1.0
Fat mass index (kg/m^2^)	9.1 ± 3.7	10.2 ± 3.9	7.4 ± 3.0*
% Body fat	33.7 ± 9.3	35.8 ± 9.1	29.2 ± 8.1*
ECW/TBW ratio (%)	40.1 ± 1.6	40.1 ± 1.6	40.3 ± 1.7
ECW/Height (L/m)	8.6 ± 1.8	8.6 ± 2.1	8.5 ± 1.2
HGS index	1.7 ± 3.5	17.1 ± 3.5	17.3 ± 3.7
ECW/height (L/m)	8.6 ± 1.8	8.6 ± 2.1	8.5 ± 1.2

*Note:* Patients divided according to dietary recall dietary protein intake (DPI). Data displayed as integer, percentage, mean ± standard deviation, or median (interquartile range). *p < 0.05, ***p < 0.001 versus lower dietary protein intake.

Abbreviations: ALMI, appendicular lean mass index; ECW, extracellular water; FFMI, fat free mass index; HGS, hand grip strength; LMI, lean mass index; TBW, total body water.

**FIGURE 1:**
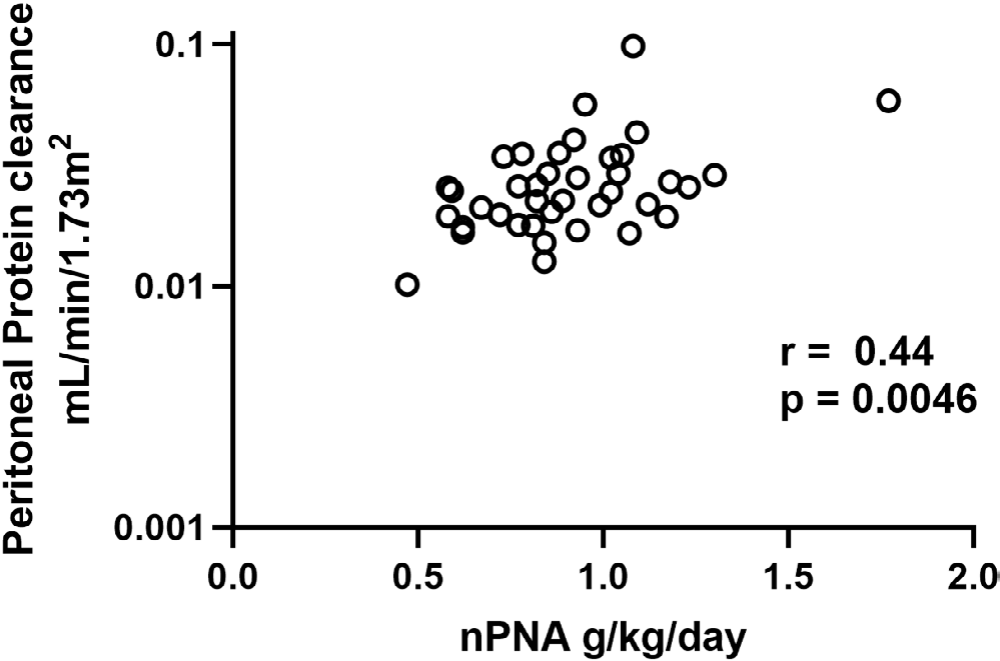
Spearman univariate association between dietary recall protein intake and that estimated from combined 24‐h peritoneal and urinary urea excretion, using standard equations [19, 20].

DPI assessed by diet recall was associated with that calculated from peritoneal and urinary urea losses, when both indexed (Figure 1), and absolute amounts (*r* = 0.48, *p* = 0.001). DPI, both when indexed for weight, and total DPI were most positively associated with dietary calorie intake, and both were associated with peritoneal protein clearance rate, protein PSTR, and 24‐h peritoneal protein loss (Table 4). In addition, both were negatively associated with ꞵ2‐microglobulin (ꞵ2M). As expected, total dietary protein was associated with assessments of muscle mass, and indexed DPI was negatively associated with indexed fat mass.

**TABLE 4:** Univariate analysis of variables statistically associated with dietary recall dietary protein intake.

Variables	Diet protein (g/kg/day)		Variables	Diet protein (g/day)	
	*r*	*p*		*r*	*p*
Calorie intake (kcal/kg/day)	0.51	< 0.001	Calorie intake (kcal/day)	0.59	< 0.001
Peritoneal protein clearance rate	0.51	< 0.001	Peritoneal protein clearance rate	0.49	0.001
PSTR protein	0.47	0.002	PSTR protein	0.46	0.003
ꞵ2 microglobulin	−0.38	0.015	ꞵ2 microglobulin	−0.46	0.003
Fat mass index	−0.37	0.016	Peritoneal protein loss	0.46	0.003
CCPD cycler versus CAPD	−0.36	0.022	Urine volume	0.41	0.007
Serum total protein	0.37	0.029	Fat free mass (kg)	0.35	0.023
Hypertonic dialysate	−0.33	0.033	Appetite score	−0.34	0.033
Peritoneal protein loss	0.33	0.035	NTproBNP	−0.34	0.028
			Co‐morbidity score	−0.34	0.029
			*K*_*t*_/*V*_urea_	0.32	0.040
			Muscle mass (kg)	0.31	0.042

Abbreviations: CAPD, continuous ambulatory peritoneal dialysis; CCPD, ambulatory peritoneal dialysis with a daytime exchange; hypertonic, 22.7 g/L glucose dialysate; *K*_*t*_/*V*_urea_, weekly combined peritoneal and urinary urea clearance; NTproBNP, N terminal probrain natriuretic peptide; PSTR, peritoneal solute transfer rate.

In a multivariable step‐backward logistic model, achieving the current recommended target DPI, then only protein PTSR remained an independent factor (4 h PSTR × 10^2^, Nagelkerke 0.36, odds ratio 7.35%, 95% confidence interval 2.5–324). In a separate step‐backward multivariable model (Nagelkerke *r* ^2^ = 0.60) DPI was independently associated with calorie intake (*β* 0.02, standard error *β* (StE β) 0.004, standardized coefficient (Stꞵ) 0.56, *t* 5.2, 95% confidence limits (CI) 0.01–0.03, *p* < 0.001), peritoneal protein clearance rate (*β* 8.48, StE 2.51, Stꞵ 0.36, t 3.4, 95% CI 3.38–0.13.57, *p* = 0.002), use of hypertonic (22.7 g/L) glucose dialysates was also retained in the model, but was not significant (*β* −0.014, StE 0.008, Stꞵ −0.19, *t* − 1.8, 95% CI −0.03 to 0.002, *p* = 0.084).

## 4 Discussion

Although KDOQI recommends that ideally dietary assessments should be made over 3 days, many elderly ESKD patients are not able to accurately recall their food intake [3]. As such, KDOQI has recommended that dietary assessments can be estimated based on dialysate and urinary urea losses. We found a correlation between dietary assessment of DPI and urea losses, both when indexed for weight and for absolute amounts. DPI and calorie intake were highly correlated. However, only around 30% of patients achieved current recommended dietary protein and calorie intake targets, and PD patients with lower DPI have been reported to have greater mortality [21]. There have been several factors proposed for why ESKD patients fail to achieve dietary targets, ranging from not changing their diet from a low protein diet on transitioning to dialysis to ongoing dietary restrictions, reduced sense of smell and taste of food, and in PD patients the physical presence of intra‐abdominal fluid, reflux oesophagitis, and glucose absorption from dialysate [22, 23]. There was an association between lower total dietary protein and worse appetite scores using a visual analogue tool.

Both total dietary recall DPI and when adjusted for body weight were negatively associated with β2‐microglobulin (β2M). Although total DPI was associated with PNA estimated from peritoneal and urinary urea losses, adjusting DPI for weight was not associated with nPNA. As urea losses are used to estimate DPI [17], to exclude collider bias we showed that there was an association between diet recall DPI and that estimated for urea‐based equations. However, DPI decreased with increasing β2M, suggesting that loss of kidney function [24], with accumulation of middle‐sized uremic toxins, rather than urea suppressed DPI.

Total DPI was associated with assessments of lean body mass, greater urine output and negatively with self‐reported poor appetite and increasing N terminal brain natriuretic peptide (NTproBNP) and co‐morbidity. However, when adjusted for weight, DPI was no longer associated with assessments of lean body mass but negatively with fat mass and increasing use of hypertonic glucose dialysates and use of a peritoneal cycler. Patients with relatively more body fat may consume less protein and calories as their increased fat stores provide additional energy, so they are less likely to need to eat as much to meet their energy needs, as a patient of the same weight but with lower fat content. More hypertonic glucose dialysates were used by patients using a PD cycler. Hypertonic glucose dialysates are used to increase ultrafiltration and so would increase the volume inside the peritoneal cavity, so potentially reducing appetite by external pressure on the stomach [22, 25]. In addition, the use of hypertonic glucose is reported to increase peritoneal glucose absorption [26], which could potentially then reduce appetite [22].

Both total DPI and when adjusted for body weight were associated with both faster peritoneal protein transport and daily protein losses, with higher DPI reported by patients with faster peritoneal protein transport. Previous studies on peritoneal protein transport have suggested that faster transport is associated with inflammation, heart failure, particularly right‐sided heart failure, and a vasculopathy [27, 28]. However, we found no association between protein PTSR and markers of inflammation, such as C reactive protein, or echocardiography cardiac ejection fraction, NTproBNP, or bioimpedance assessments of increased extracellular water to total body water or height ratios. Although peritoneal protein losses might suggest an increased risk for a negative nitrogen balance and muscle wasting, observational studies have not shown an effect on muscle wasting [29]. Indeed, we noted that although DPI was associated with faster protein PSTR and greater protein losses, it was also associated with higher total serum protein. As such, our results would suggest that the normal, but complex and highly regulated feedback loop between protein losses and DPI is maintained in PD patients [30].

## 5 Strengths and Limitations

We report associations between DPI, body composition, muscle strength, physical activity, and additional co‐morbidities in this cross‐sectional analysis, and as such we cannot attribute causality. To avoid inter‐observer differences in obtaining dietary histories and measurement of both HGS and bioimpedance measurements of body composition, particularly when peritoneal dialysate was not drained from the abdomen, all measurements were performed by a trained specialist renal dietitian, who was blinded to the results of peritoneal dialysis adequacy and PTSR. Diet recall can be affected by memory lapses, so patients with dementia, learning disorders and a history of previous stroke and cerebrovascular vascular disease and those with a language barrier were excluded [31]. As such, we acknowledge the sample size of patients completing food recalls; however, this reflects the size of a single center PD population in Europe. To limit errors in recall, self‐reported dietary intake was limited to the previous 24 h, as other studies have highlighted the difficulty that elderly co‐morbid patients have in accurately recalling or documenting their food intake over longer time periods. To estimate portion sizes, patients were shown a series of visual illustrations.

## 6 Conclusion and Implications

Whereas studies in the general population have reported an association between DPI and estimates of lean body mass [2], we found no such association in our cohort of ESKD patients treated by PD. A previous study from more than a decade ago limited to PD patients treated only by CAPD reported an association between peritoneal protein clearance measured using the biuret method, which is recognized to be inaccurate at low protein concentrations and lean body mass [32], however, their patients with greater peritoneal protein losses had both a lower serum albumin and total protein along with a lower estimated DPI. We report that PD patients with greater peritoneal protein losses had faster protein PSTR and greater DPI. As such, the regulatory mechanisms linking protein loss and catabolism to DPI function to some extent in PD patients. Even so, the majority of PD patients failed to achieve the current recommended protein DPI targets, and, as there was a strong association between protein and energy DPI, equally failed to achieve both targets. As such, we would advocate that peritoneal protein losses should be measured and taken into account when advising dietary intake.

## Funding

The authors have nothing to report.

## Ethics Statement

UK National Health Service (NHS) Research Authority Ethics Service (NRES) (EDGE 158772, IRAS29331).

## Consent

In keeping with the Helsinki accord, all patients provided written informed consent.

## Conflicts of Interest

The authors declare no conflicts of interest.

## Data Availability

The data that support the findings of this study are available on request from the corresponding author. The data are not publicly available due to privacy or ethical restrictions.
